# Current context and evaluation of medical residency programs: experience of six Brazilian Medical Societies

**DOI:** 10.1590/0100-6991e-20243861-en

**Published:** 2024-12-12

**Authors:** GERSON ALVES PEREIRA, RAMIRO COLLEONI, JAIR GIAMPANI, JORGE CARVALHO GUEDES, REGINALDO RAIMUNDO FUJITA, ADRIANO FERNANDO MENDES, GUSTAVO SALATA ROMÃO, LUCAS NEPOMUCENO BARROS, LUIZ ALONSO DAVID, PAULO FERNANDO CONSTANCIO DE SOUZA, JOSÉ EDUARDO LUTAIF DOLCI, CESAR EDUARDO FERNANDES

**Affiliations:** 1 - Universidade de São Paulo, Campus Bauru - SP - Brasil. Presidente da Comissão do Título de Especialista do Colégio Brasileiro de Cirurgiões.; 2 - Universidade Federal de São Paulo - SP - Brasil. Presidente da Comissão de Residência Médica do Colégio Brasileiro de Cirurgiões.; 3 - Universidade Federal de Mato Grosso - Cuiabá - MT - Brasil. Coordenador da Comissão de Ensino do Conselho Brasileiro de Oftalmologia.; 4 - Universidade Federal da Bahia - Salvador - BA - Brasil. Coordenador da Comissão do Título de Especialista da Federação Brasileira de Gastroenterologia.; 5 - Universidade Federal de São Paulo - SP - Brasil. Comissão de Ensino e Treinamento da Associação Brasileira de Otorrinolaringologia e Cirurgia Cérvico-Facial.; 6 - Universidade Federal de Juiz de Fora - MG - Brasil. Comissão de Ensino e Treinamento da Sociedade Brasileira de Ortopedia e Traumatologia.; 7 - Universidade de Ribeirão Preto - SP - Brasil. Presidente da Comissão de Residência Médica da FEBRASGO.; 8 - Secretário da Comissão de Certificação em Anestesiologia CCA/SBA. Diploma Europeu de Anestesiologia e Terapia Intensiva, EDAIC/ESAIC.; 9 - Universidade de São Paulo - São Paulo - SP - Brasil.; 10 - Comissão Estadual de Residência Médica de São Paulo (CEREM/SP), Câmara Técnica de Ginecologia Obstetrícia do Conselho Regional de Medicina de São Paulo - São Paulo - SP - Brasil.; 11 - Faculdade de Ciências Médicas da Santa Casa de São Paulo - SP - Brasil. Diretor Científico da Associação Médica Brasileira (AMB).; 12 - Faculdade de Medicina do ABC - Santo André - SP - Brasil. Presidente da Associação Médica Brasileira (AMB).

**Keywords:** Training of Human Resources in Health, Residency, Competency-Based Education, Evaluation of Human Resources in Health, Capacitação de Recursos Humanos em Saúde, Residência Médica, Educação Baseada em Competências, Avaliação de Recursos Humanos em Saúde

## Abstract

This article shows the most recent data on the evolution of the number of resident physicians and vacancies from R1 to R5, as well as the number of programs and institutions accredited by the National Commission for Medical Residency (CNRM). It also discusses the types and modalities of evaluation of medical residency, with a focus on the assessment of competencies throughout in-service training, which were incorporated into the most recent CNRM resolution, at the end of 2023, which amended and updated the 2006 directives. Finally, it shows the experience of six Medical Societies that conduct periodic evaluation of resident physicians, presented at a Brazilian Medical Association event.

## RESUMO

Este artigo mostra os dados mais recentes da evolução do número total de médicos residentes e vagas de R1 a R5, bem como do número de programas e instituições credenciadas pela Comissão Nacional (CNRM). Também discute os tipos e modalidades de avaliação da residência médica, com foco na avaliação das competências ao longo do treinamento em serviço, que foram incorporadas na mais recente resolução da Comissão Nacional de Residência Médica (CNRM) do final de 2023, que alterou atualizou as determinações de 2006. Por fim, mostra a experiência de seis Sociedades Médicas que fazem a avaliação periódica dos médicos residentes que foi apresentada em evento na Associação Médica Brasileira.

## INTRODUCTION

Internationally recognized as the most appropriate modality for training specialists, Medical Residency (MR) is defined as postgraduate education, characterized by intensive and in-service theoretical-practical training, under the supervision of medical professionals with high ethical and professional qualifications (BRASIL, 1981).

Although it is not mandatory for the practice of the profession in Brazil, residency is considered the “gold standard” of medical specialization. Medical Residency Programs (MRPs) grant the Specialist Title (ST). The other way to obtain a ST is by fulfilling the criteria to apply for a specialist title exam of a medical society associated to the Brazilian Medical Association (AMB). These are the two types of titles accepted by law (BRASIL, 2015) for a doctor to obtain the Specialist Qualification Registration (SQR), a document that proves that a doctor is qualified to work in a medical specialty.

It is the responsibility of the National Commission for Medical Residency (CNRM), of the Ministry of Education (MEC), to manage the accreditation and inspect the operating conditions of the institutions that offer MRPs, as well as to authorize, evaluate, and renew the programs, with the participation of the State Medical Residency Commissions (CEREMs).

The admission of physicians to MRPs occurs through a public call, via public notices and selection processes. The duration of programs in medical specialties varies from two to five years, while MR in some medical areas can add one or more years of training.

The resident doctor receives a monthly scholarship, whose value, in 2024, is R$ 4,106.09 (~ US$ 707.94 - exchange rate of 5.8 BRL/USD) for a special in-service training regime of 60 hours per week.

The financing of MR in Brazil is mostly public. The Ministry of Health (MH) is the main payer, providing for about 40% of the MR scholarships, followed by the Ministry of Education and state governments, in addition to the participation, on a smaller scale, of city halls and philanthropic and private hospitals (SCHEFFER et al, 2023).

The most current panorama showed that 47,700 doctors were attending Medical Residency (MR) in 2024. These resident physicians represented about 8% of the physicians in the country. A total of 19,551 physicians were enrolled in R1, the first year of Medical Residency. The number of accredited Medical Residency Programs (PRM) was 5,631 in 2024, comprising the 55 specialties and 62 recognized areas of activity (BRASIL, 2024) recognized by the Joint Committee of Specialties (CME), composed of representatives of CNRM, the Federal Council of Medicine (CFM), and AMB (SCHEFFER et al, 2024). 

**Figure 1:**
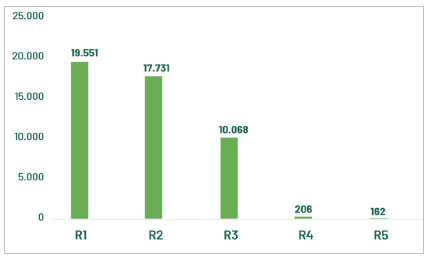
Resident physicians in Brazil, according to the current MR year (R1 to R5), in 2024. Source: CNRM/Sesu/MEC; Scheffer, M. et al. Medical Demography in Brazil. Note: R1 vacancies include those with direct access and those requiring prerequisites.

Figure 1 shows the general distribution of resident physicians categorized by current period (year) in the Medical Residency Programs in 2024.

**Figure 2:**
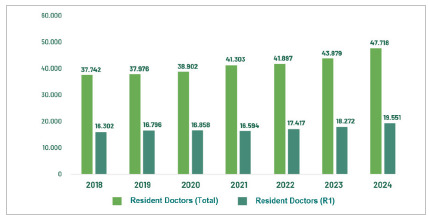
Evolution of physicians attending medical residency and physicians in the first year (R1), from 2018 to 2024. Source: CNRM/Sesu/MEC; Scheffer, M. et al. Medical Demography in Brazil. Note: R1 vacancies include those with direct access and those requiring prerequisites.

Annually, there has been a decrease in the demand for first-year vacancies (R1), as can be seen in Figure 2.

**Figure 3:**
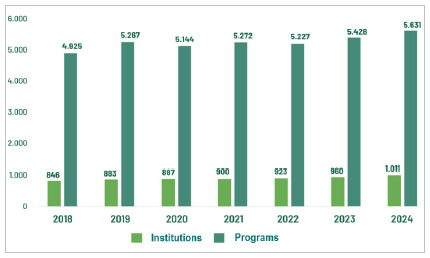
Evolution of MR institutions and programs, from 2018 to 2024. Source: CNRM/Sesu/MEC; Scheffer, M. et al. Medical Demography in Brazil.

The number of institutions offering MR has increased by about 20% in the last 8 years, from 846 in 2018 to 1,011 in 2024. The number of programs grew by about 14%, from 4,925 in 2018 to 5,631 in 2024, with oscillations over the years (Figure 3).

A total of 386 programs and 55 institutions were identified that, although accredited by the CNRM, did not have, in 2024, any MR vacancies filled.

Regarding the evaluation of resident physicians, it is up to the program to ensure that the graduate reaches the desired level of competence (MEGALE et al, 2009). As it is an in-service training, evaluating in this context goes beyond cognitive analysis, being a daily challenge for the professor and the preceptor. 

The conceptual model proposed several decades ago, known as Miller’s pyramid (MILLER, 1990), demonstrated to professors that, in professional development, evaluation cannot be restricted to theoretical knowledge, as it is necessary for the student to know how to apply this knowledge, execute it in a practical way, in simulated environments, and finally, apply it in real life (SOUZA, 2012). 

The implementation of the competency-based model implies the employment of measures by evaluation methods. “Knowing” and “knowing how” are domains of cognitive competences, which is the theoretical knowledge of the professional. The following domains, on the other hand, bring together the competencies related to the practical application of the acquired knowledge. “Show how” indicates what the professional can do and allows one to know their clinical skills. This includes psychomotor (physical examination, medical procedures, etc.) and behavioral (communicating with patients and colleagues, complying with patient safety procedures, etc.) competencies. “Doing”, on the other hand, describes the real performance of physicians, that is, how they really perform their functions in the midst of the pressures of a dynamic environment, with its uncertainties and subjectivities, their ethical behavior in the face of emotionally complex situations, and flexibility in the face of diverse demands (BOURSICOT et al, 2011). 

**Figure 4:**
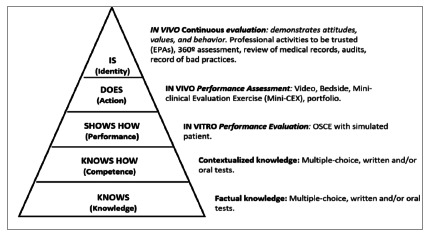
Updated version of Miller's Pyramid (1990 ), with examples of what forms of evaluation should be used at each level. Adapted from Cruess et al., 2016 .

Miller’s pyramid aligns its strata with educational objectives and evaluative methods directed to the types of ability and competence whose domain is to be known, ascending from the theoretical knowledge contained in the base - “knowing” and “knowing how” - to “show how” and “doing” (Figure 4). The apex of the pyramid corresponds to the evaluation of professionals in their work environment (PANUNCIA-PINTO & TRONCON, 2014). 

For the learning objectives that deserve to be evaluated, the concept of performance was adopted, that is, the actions effectively manifested, in which the student should achieve a given performance, defining the level considered acceptable (Gontijo et al., 2013). The best way to evaluate performance is to define the task to be held in each context and to verify students’ ability to mobilize their attributes in that situation in an appropriate way to perform the task (Aguiar & Ribeiro, 2010). Thus, the choice of assessment methods requires clarity of objectives, as well as knowledge of their psychometric properties to identify which ones should be used at each moment (Wass et al., 2007).

The implementation of the competency-based model (Epstein & Hundert, 2002) implies the measurements by evaluation methods (Norcini et al., 2013). A widely used approach to facilitate such measures was proposed by Miller, who grouped similar competencies into broader domains. Figure 4 shows the updated version of Miller’s Pyramid (Miller, 1990; Boursicot et al., 2011 and Cruess et al., 2016), with examples of what forms of assessment should be used at each level.

[Fig f5]

**Figure 5: f5:**
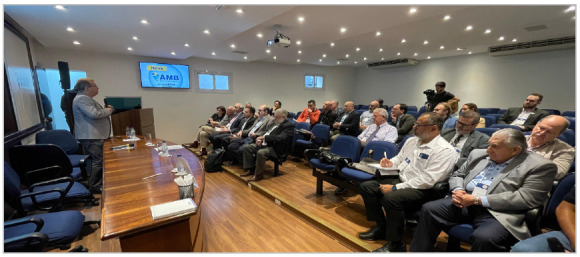
Photo of the AMB event on “Brainstorming of medical residency evaluation models” - November 18, 2024.

Based on these dimensions and the degree of learning of the resident physician, the observations made by professors and preceptors should be directed, in addition to the cognitive, to performance evaluations, considering clinical and psychomotor skills, interaction with the patient, information management, judgment, synthesis, and decision-making capacity, as well as the preservation of ethical attitudes (MEGALE et al, 2009). 

The importance of performance appraisal at this level of training is due to its potential to verify clinical skills (communication, physical examination, and procedures), and continuous and formative evaluation, allowing the correction of failures and reducing the possibility of errors (AUTO et al, 2021). 

Within the psychometric perspective, the subjectivity of the evaluators and the specificity of the clinical cases in the practical tests are highlighted, which potentially interfere in the performance evaluation results of students, residents, or candidates. (2) This difficulty of professors and preceptors in evaluating and formulating tests due to the lack of theoretical basis, in addition to the absence of standardization, has been very noticeable in practice and has been shown in several studies (ZIMMERMANN et al, 2019; AUTO et al, 2021).

The improvement of the professor and the preceptor in evaluation ensures the quality of the assessment and the teaching-learning process, since the evaluation allows the review of the educational planning and adjustments in their teaching practice (ZIMMERMANN et al, 2019). This is a limiting factor in most evaluations.

Most of the clinical skills assessment methods have, as a basic principle, the direct observation of the resident’s performance in clinical tasks, in a real or simulated environment. In this sense, it should allow feedback, preferably immediate (formative), which consists of describing and discussing with the residents their performance in each activity (ZEFERINO & PASSERI, 2007).

With clinical simulation, multiple competencies can be tested simultaneously: patient care, medical knowledge, psychomotor skills for procedures, professionalism, and interpersonal skills, among others, allowing both the development and evaluation of individual competencies in similar situations that occur on a daily basis and effective team collaboration and the construction of a safety-oriented culture (Pereira Jr., 2021).

Since its 2022 edition, the evaluation process of candidates for the Specialist Title of the Brazilian College of Surgeons has, in the second phase (practical test), a practical test using simulated stations, both face-to-face and online, complementing the oral test with discussion of clinical cases, (Pereira Jr et al, 2024).

To make the assessment of medical competencies a more comprehensive, valid, and reliable process, instruments with predefined criteria were developed, both for a simulated environment, such as the Structured Clinical Objective Examination (OSCE), and for day-to-day practice environments, such as the Mini-Evaluative Clinical Exercise (Mini-CEX) and the Direct Observation of Procedural Skills - DOPS (Norcini et al, 2003; Wilkinson et al 2008; Kogan et al, 2009). 

Despite the valuable contribution of simulation-based education, it should not be forgotten that its various modalities do not replace clinical experience and that, like any other intervention in Medicine, its use must be supported by evidence, as there are risks when students are appraised with only certain types of simulators to the detriment of real patients. Despite the numerous advantages, it is essential to check whether these benefits translate into clinical practice. Regarding the execution of procedures, it has been shown that the volume of experience reduces complication rates in patients, improving performance even before these procedures are conducted in real practice settings (DOMURACKI et al., 2009). 

Research on simulation-based education often does not address the most relevant question of predictive validity, i.e., whether the capabilities acquired in a simulated environment can be generalized to real clinical situations. Since there is difficulty in designing and executing studies that allow verifying this hypothesis, since it would require long-term follow-up of students, the transfer of this learning from simulation to clinical and surgical practice remains to be understood. Thus, it is essential to recognize that this methodology may not be appropriate in all cases, establishing which simulation use cases will bring advantages. It is also necessary to clarify whether the learned skills are maintained for a lengthy period (Pereira Júnior, 2021).

Because performance in a controlled or simulated environment does not reliably predict performance in clinical practice, work-based assessment (WBA) has become indispensable and has been rapidly incorporated into residency programs around the world. In the United Kingdom, for example, WBA is used in all programs recognized by the Royal College (Crossley & Jolly, 2012). 

As advantages, these instruments are easy to use, do not interfere with the service’s routine, enable the identification and correction of deficiencies during the training period, and are intended for the evaluation of the higher levels of the Miller pyramid (Norcini, 2005; Holmboe, 2017).

There are some limitations related to this type of evaluation. Regarding validity, this type of approach can be “reductionist” in assessing all the complexities of the physician’s professional conduct (Crossley & Jolly, 2012). Regarding reliability, the psychometric analysis showed highly variable results, with a significant difference between evaluators, with a tendency to classify most of the trainees very positively (Kogan et al, 2009). Given these limitations, WBA has been used primarily as a formative assessment, although in some cases it can also be included as one of the components of summative assessment (Ponnamperuma, 2013). To ensure WBA validity, it is recommended to broaden the observation spectrum of the cases treated with a sample that represents the true diversity of the resident’s practice. To ensure WBA reliability, the number of observers should be expanded and, if possible, include other perspectives in addition to the preceptors, such as their peers, other team members, and the patients they care for (Ponnamperuma, 2013).

A well-designed and periodic evaluation system, with continuous feedback, is an effective tool to improve the performance of future specialists and ensure their qualification, a goal of indisputable importance in the training process. To this end, resident assessment needs systematization and institutionalization as to how to evaluate, in addition to teacher training for this important aspect of the teaching-learning process (AUTO et al, 2021). 

There are several options for assessment in clinical practice environments, which can be subdivided into two evaluation methods: 1) based on observation of performance in practice, by a single observer or multiple ones (a- Clinical Care - Clinical Evaluative Mini-Exercise (Mini-Cex), b- Case Based Discussion (CbD), c- Professionalism - Professionalism Mini-Evaluation Exercise (P-Mex), d- Procedures - Direct Observation of Procedures (DOPS), and Objective Structured Assessment of Technical Skills (OSATS) and Non-Technical Skills for Surgeons (NOTTS); and, 2) based on documents and records (Logbook - Logbook and Portfolios) (Romão et al, 2020).

Competency milestones are narrative descriptions of behaviors in a continuous progression of the learners’ development, in which they start as beginner students and advance throughout training until they become competent and can reach the level of experts (after years of deliberate practice) (PEREIRA JÚNIOR et al., 2015).

The use of competency frameworks exceeds all the expectations of learners, as it makes them aware of what they need to perform, from the initial stages of training to the final supervised educational practices, when they are expected to obtain specialization in a certain competence to meet the needs of health care (ACCREDITATION COUNCIL FOR GRADUATE MEDICAL EDUCATION; AMERICAN BOARD OF EMERGENCY MEDICINE, 2013).

The learners’ progress is measured, and it is possible to determine whether there has been progression, stagnation, or regression in development as an indication of the absence or specific needs for corrections. The competence framework is equivalent to the behavior observable within five levels of proficiency, and through them students demonstrate the knowledge, skills, attitudes, and performance that reflect each of these development levels (PEREIRA JÚNIOR et al., 2015).

These narrative descriptions of learners’ behavior have been effective in organizing more complex and large actions, allowing the possibility of formative feedback to stimulate changes in observed behaviors, in addition to enabling greater precision in the application of scales and evaluation strategies (LOMIS et al., 2017).

However, the descriptions of the competencies are contextual and independent, so they do not define the complexity of implementation in a clinical setting, in which the competencies will be demonstrated (TEN CATE, 2013). Thus, Olle Ten Cate sought to bridge the gap between theory and clinical practice, presenting the Entrustable Professional Activities (EPAs), internationally recognized as a framework for significant assessment of competence during undergraduate training, medical residency, and professional recertification (TEN CATE, 2013).

EPAs represent a unit of professional practice that can be entrusted to a student or competent professional and that require simultaneous proficiency in multiple competencies. This approach provides a broad and practical approach to assessment, since it does not evaluate competencies individually or in isolation (TEN CATE, 2013).

EPAs are tied to assignment decisions, i.e., they assess whether the apprentice can perform certain clinical practice activities under a designated level of supervision. Apprentices can be entrusted with the responsibilities or tasks that must be done in patient care, which can be small or large, that is, simple or complex, but are usually activities with a beginning and end, and are entrusted only to competent people (TEN CATE, 2018).

Thus, EPAs are characterized as an evolution of the competency-based educational concept, in which the concept of a learner’s competencies is applied in specific contexts in the clinical practice site, constituting a job description and being independent of people (TEN CATE et al., 2015).

## Evaluation of Medical Residency Programs in Brazil

The residents’ evaluation process had a traditional and summative character, although CNRM defined guidelines for the evaluation of resident physicians since its resolution no. 05, of November 12, 1979 (BRASIL, 1979), reiterated in CNRM resolution no. 02, of May 17, 2006 (BRASIL, 2006).

CNRM published in the Official Gazette of the Union a new resolution (Resolution No. 4, of November 1, 2023) that regulates and clarifies the procedures for evaluating resident physicians in medical residency programs authorized and offered by Institutions accredited to the federal agency, repealing articles 13, 14, 15 and 16 of Resolution No. 2, of May 17, 2006.

Chart 1 shows a comparison between the CNRM previous and current resolutions

**Chart 1:** Changes in CNRM resolutions on the evaluation of resident physicians. Source: authors.

ITEM	PREVOUSLY (2006)	CURRENTLY (2023)
Assessment frequency	Quarterly	Every four months
Responsibility for residence supervision	Professors with a Medical Residency certificate in the specialty area, or with a higher degree, or with equivalent qualification, according to COREME	Supervisor and set of preceptors; joint evaluation between programs from other institutions in the same modality is prohibited
Evaluation methods	Written, practical, or performance test by attitude scale	Multiple methods and instruments of summative and formative assessments in 3 modalities: 1) Cognitive (theoretical), 2) Psychomotor (practical), and 3) Affective-Professional. Structured feedback
Evaluation concepts	Post-Evaluation: concept of sufficiency defined by COREME by the average value of the conducted evaluations	Pre-Assessment: concept of sufficiency defined in the preparation of the assessment, representing the expected performance for the evaluated domains
Can be included in the Evaluation System by COREMEs	Monograph and/or presentation or publication of a scientific article at the end of training.	- Record of procedures and activities: Logbook, portfolio, and Scientific Research during training; - Progress Test (120 to 200 questions) prepared by the Specialty Society.
Resident Approval for the following year	- Full compliance with the program’s workload; - Approval by the average value of the evaluations conducted at the discretion of COREMEs.	- Full compliance with the workload and evaluations of the program; - Mandatorily achieve a minimum score of 7 in the 3 cognitive evaluations; - “Satisfactory” concept of the quadrimester evaluations in the psychomotor and affective-attitudinal components; - EPAs could serve as bases for verifying progress at the levels of supervision and autonomous practice.
Program Certification	- Full compliance with the program’s workload; - Approval by the average value of the evaluations conducted at the discretion of COREMEs.	- Full compliance with the program’s workload; - Full compliance with the criteria of periodic evaluations; - Full compliance with the promotion criteria in all years of residency; - Presentation of the final work for the conclusion of the course

It is known that the Medical Residency Committees (COREMEs) have not been able to satisfactorily meet the evaluation goals that were determined. Without any help, these changes in the evaluation procedures of resident doctors will only be “notary”. This help could come from the Specialty Societies.

Some progress items in the above changes regarding the performance evaluation of resident physicians must be permanent and continuous, considering knowledge, acquired skills, and professional attitudes. Its main objective will be to prove the professional’s learning evolution throughout this modality of medical training. Thus, a set of assessments is recommended that involve areas such as technical knowledge, decision-making, professionalism, ethics, relationship with the team, patients and family members, as well as performance in the health system. The commitment and development of the curricular activities established in the Competency Matrices of the respective programs should also be evaluated, highlighting the need for feedback at the end of the evaluations, pointing out both the positive aspects and the points for improvement.

For this, assessments should be both summative (cognitive - theoretical - and psychomotor - practical) and formative (Affective-Professional - Attitudinal Evaluation in Professional Practice Environments). The latter should be evaluated through direct and indirect observation of the resident physician’s performance by the preceptor, group of preceptors, and supervisor, considering responsibility, attendance, punctuality and fulfillment of tasks, performance in the dynamics of the program, collaboration with the construction of knowledge, communication, and interpersonal relationships. For this, if possible, it should also include evaluation by peers, team members, and patients.

A major difficulty has been the approach to resident physicians with unsatisfactory performance. The new resolution defines that the resident who does not obtain a minimum average of 7.0 (seven) in each of the three annual training evaluations will not be considered able to advance to the following year. In addition, the resident who does not perform satisfactorily in the evaluations in professional practice environments after the conclusion of the annual training period will not advance to the following year. Finally, the resident physicians with insufficient performance at the end of the annual training period, even after a retake exam, will be dismissed, regardless of the year they are attending.

The Individual Resident Progress Test prepared by the respective specialty society may also be adopted as a complement to the evaluation and progression process, at the discretion of COREME. This should be held annually and simultaneously for residents of the same program and will consist of 120 to 200 multiple-choice questions addressing the content presented during the program and according to the established Competency Matrix.

In this way, in addition to the four-monthly internal evaluation of the medical residency programs (PRMs) themselves, there would also be a periodic external evaluation by the respective Specialty Society, reducing the risk of endogeny and showing a national overview of the quality of training in medical residency, which will provide important information on the strong points and those that need to be improved.

In view of this current context, the Brazilian Medical Association (AMB), in partnership with the São Paulo Chapter of the Brazilian College of Surgeons, organized a pilot event, on November 18, 2024, about this discussion called “Brainstorm of medical residency evaluation models”, inviting the heads of six specialty societies with experience in this activity (Brazilian Association of Otorhinolaryngology and Craniofacial Surgery - ABORL-CCF, Brazilian Council of Ophthalmology - CBO, Brazilian Federation of Gastroenterology - FBG, Brazilian Society of Anesthesia, Brazilian Federation of Gynecology and Obstetrics - FEBRASGO, and Brazilian Society of Orthopedics and Traumatology - SBOT) and the CNRM Executive Secretary.

The wealth of stories and experiences of each of the Medical Societies in the construction of their periodic evaluations of medical residencies is very interesting, in relation to: 1) year of creation of the Society; 2) year of start of the specialist title exam; 3) year of start of the resident evaluation exam; 4) differences in the application of the evaluation between R1, R2, and R3; 5) types of assessment (Cognitive - multiple-choice tests, discursive; 6) Oral - discussion of clinical cases; 7) face-to-face or online simulated stations, and other types of assessment; 8) frequency of application of the tests; 9) how to prepare each test; 10) approval criteria; form of selection and training of evaluators; 11) form of dissemination of results; 12) form of feedback to residents and services; 13) way of approaching the appeals and questions about the various types of evidence; 14) offer of a training course for resident doctors; 15) offer of a training course to preceptors; 16) presence of a residence certified by the Society itself (non-MEC); 17) mandatory end-of-course work/publication; 18) scoring of the medical residency tests for the Society specialist title exam; 19) limitations not yet resolved; 20) validity time and recognition of the title; and 21) form of revalidation/recertification of the title.

The final proposal discussed is to plan an event in the first half of 2025, inviting all 54 Specialist Societies and 27 Federated Societies of the Brazilian Medical Association and expanding the topics to be addressed: 1) Sequential evaluation of the continuing education process of medical training (undergraduate studies, medical residency and specialist title); 2) Evaluation of services; 3) Financing of medical residency scholarships; 4) Strategies for filling vacant positions; 5) Legal support; 6) Theoretical-practical program of PRMs; 7) Technical and pedagogical training of preceptors, 8) Training of supervisors and preceptors of PRMs for the various types of summative (cognitive - theoretical and psychomotor - practical) and formative (Affective-Professional - Attitudinal Assessment in Professional Practice Environments) assessments; 9) Training of those responsible for PRMs for feedback; 10) Quotas in medical residency; 11) Exchange of resident doctors between programs and 12) Mental health of residents and preceptors, among others.

Table 1 below compares the main information and indicators about the periodic evaluation of the medical residency programs of the six Medical Societies that participated in the event.

**Table:** 

INDICATORS	CBO	SBOT	FBG	SBA	ABORL/CCF	FEBRASGO
Year of Society's creation	1941	1935	1949	1948	1948	1959
Starting year of specialist title exam	1986	1970	1957	1983	1971	1967
Starting year of resident assessment exam	1986	1972	2024	1983	2012	2018
Differences in exam applications for R1, R2, and R3?	Currently none.	Before 2025, two assessments: a simulated one - TARO - applied to all years; and the title exam - TEOT - only for R3. From January 2025 - TEPOT - for R1 and R2	There is not, as a progress test is applied	Obtaining the title includes quarterly tests and an annual test. The quarterly tests are mandatory, address topics of each quarter, prepared by the Anesthesiology Certification Commission (CCA). The quarterly tests will be held exclusively online remotely at the CET. The physician in specialization (ME) who does not submit to the quarterly test, due to force majeure, may, through the Person in Charge of the CET who is studying, request the performance of a substitute test, on the date established by SBA, after sending the pertinent original documentation. If authorized by CCA/SBA to take the substitute test, it must be applied in February of the current academic year and will be conducted in electronic format (online), as decided by SBA. The annual test at the beginning of each year, on a Sunday, from 10 am to 12 pm exclusively online, through a secure browser, contracted by SBA with eduCAT, installed on the candidate’s personal computer, with internet access and equipped with webcam and microphone.	The realization and application of the test are governed by the public notice under the coordination of the Specialist Title Committee, being aimed at all residents and post-graduates in Otorhinolaryngology in services accredited and recognized by ABORL-CCF. R1 and R2 get a bonus of 0.2 points if they reach the score determined by the Committee. R3 only participates in the annual exam to obtain the Specialist Title, after completing and being approved in the three years of medical residency in services accredited by the MEC or recognized by ABORL.	Single test for R1, R2, and R3, leveled by the finishers (R3)
Types of evaluation (enter year of start and number of questions/cases/stations of each): - Cognitive (multiple-choice, discursive tests) - Oral (discussion of clinical cases) - Simulated stations - Other types of assessment	200 multiple-choice questions distributed in 3 tests (Theoretical I, Theoretical II, and Theoretical-Practical) applied online to R1 and R2 (progress test) and R3 (PNO- title test)	Start year/current number of questions: - Written Cognitive Test - 1972 / 100 questions - oral - 1972 / 16 situations - Physical Examination Test - 1994 / 04 situations - Skills Test 2011/05 Situations - Attitudes in the physical examination - 2016 / 01 (one) situation - Anatomy Assessment - 2018 / 20 questions	Progress test (PT): Multiple choice test (MCQ) 120 items with 4 assertions (1 correct and 3 distractors) based on clinical situations. Title Test - MCQ with 100 questions. (70% of the grade) + curriculum analysis with Barema (30% of the grade)	15 questions for each year of MR, totaling 45 questions, with five items of V or F each question. Annual test with 120 questions, multiple choice.	Year of start: 2012. Theoretical test with Otorhinolaryngology content, lasting three hours, consisting of 100 (one hundred) multiple-choice questions with four alternatives each and only one correct option. Each question will correspond to the value of 0.1 (zero point one) point, totaling 10 points;	PROGRESS TEST THEORETICAL TEST, multiple-choice test, with 60 questions of Gynecology and 60 questions of Obstetrics based on the Competency Matrix. According to the Legislation, it is up to the programs to carry out other evaluations of the resident such as Internship Test, Oral Test, Practical Tests, including simulated stations.
Exam’s frequency of application	Annual	Annual, at the end of R3	PT: twice a year (we had a pilot test) Title test: once a year	4 quarterly exams + 1 annual exam	Annual	Annual
Exam preparation	Exam Committee, based on the competency matrix	Teaching and Training Commission, composed of 12 full members of the SBOT, who meet monthly during the year to discuss, format, and approve the exam.	Items considering thematic matrix distributed according to epidemiological relevance, classified into subareas, considering service scenarios, type of competence (pathophysiological bases, diagnosis, etc.), degree of difficulty. The matrix is the same for all tests. Shared	Test prepared by a committee appointed by the SBA.	Theoretical test with multiple choice questions.	The preparation and review of the items is done by the Specialist Title Commission (CNTEGO)
			elaboration. Title test uses the same thematic matrix.			
Approval Criteria	The candidate who obtains an arithmetic average in the Theoretical I, Theoretical II, and Theoretical-Practical Tests equal to or greater than 6.5, will be considered approved, worthy of the Specialist Title in Ophthalmology, provided that he/she also obtains a minimum score of 6.0 in each of the Tests and a minimum of 7.0 in the Practical Test.	Grade 6.0	Title test: Not considered (formative assessment)	In each year of the Specialization course, the resident must obtain a minimum average for approval equal to or greater than 6.0. The final grade of each academic year will be calculated as follows: the arithmetic average of the grades of the four quarterly evaluations conducted by the CCA (including the theoretical tests and the skills and behavioral evaluations), will be added to the grade obtained by the resident in the annual test prepared by the Anesthesiology Certification Commission - CCA.	Equal/Greater than 7	PROGRESS TEST - there is no pass or fail. The exemption and bonus in the Theoretical Test of the Specialist Title of the following year depend on the attendance and performance of the candidate in the Progress Test such as R1, R2, and R3. FOR WAIVER: participation in the 3 applications, performance greater than or above 60th percentile in at least 2 versions, including R3, and correct answers in at least 65% of the Gynecology and Obstetrics questions in R3. FOR BONUS: participation in the 3 applications, performance greater than or above 30th percentile in all versions, and correct answers in at least 50% of the Gynecology and Obstetrics questions in R3.
Evaluators Selection and training	Appointment by the Teaching Commission and Executive Board of the CBO	Recruited as observers and then become examiners.	Academic interest, teaching, choice of regionally based management	Members of the examining committee are elected in a specific assembly and submitted to training and qualification courses.	The Examining Board will be composed of members of the ABORL-CCF Specialist Title Committee.	There are no raters for the Progress Test. The test is applied online, and candidates are monitored.
Results publication	CBO Platform	List of approved published on website.	PT - confidential disclosure for candidates and services, not ranking. Title test - list of approved	The final grade of each Physician in Specialization will be made available to them and to those responsible for their CET, in a specific area on the SBA portal	ABORL-CCF communication channels	Online through the FEBRASGO platform
Form of feedback to residents and services	Coordinators have access to their students’ grades, and each student has access to their individual grades	Individualized performance report, with a copy to the program supervisor.	. Detailed performance analysis by the matrix criteria, comparative to the regional and national pool, with performance and evolution graphs	Through those responsible and in a specific area on the SBA portal.	Charter and communication channels.	The individual performance of each resident in the PROGRESS TEST is confidential, accessed by the resident through a password. The consolidated results of the residents of the same program are provided to the supervisor, provided that at least 3 residents of each category have applied to the Test
Way of approaching the appeals and questions about the various exam types	Online test applied through a specific platform contracted with a specialized company	For 2025 there will be resources according to the guidelines in the notice.	Title test- Referral of applicants to the board PT- no appeals. Local discussion by supervisors	SBA website or company hired to apply the test.	Appeals	The appeals are evaluated and responded to by CNTEGO and the FEBRASGO Residency Commission
Is there a training course offered to residents?	Yes	SBOT encourages continuing education for all residents and the Progress Test (TEPOT) is an institutional initiative to this end. SBOT supports regional subsidiaries that conduct local preparatory courses for residents.	Yes, not mandatory. Annual Gastroenterology Course, Young Gastro Program with cases and updating topics; FAPEGE (Foundation for Research Support in Gastroenterology) classes, Gastrotalk program (podcasts)	Yes.	Continuing Medical Education Platform	FEBRASGO offers online programming for updating resident doctors in the specialty (CONECTA-GO)
Is there a training course offered to preceptors?	Yes	Yes, annually the Forum of Preceptors, sessions at the Annual Congress specific to preceptors, and training and improvement courses in preceptorship have already been held.	TTT (Train the Trainers) in partnership with the World Gastroenterology Organization (WGO) with 2 training centers in Brazil (RG and CE)	Yes	Course for the Training of Preceptors.	Periodically, FEBRASGO offers a training program for preceptors
Does the Society have a residence-like course (not MEC, certified by itself)?	Yes	Yes	They are not from FGB but it accredits courses if they have a prerequisite fulfilled in Internal Medicine, theoretical, and practical workload equal to that of MR	Yes	Specialization 3 years	No
Does the Society require an end-of-course completion work/publication? Does this have weight in the evaluation?	Can occur in each course separately but is not a CBO requirement	Yes, to register for the title test, it is necessary to send a scientific work, which is mandatory. A note of this item is provided for in the notice.	Not applicable	Yes	No	No
Does the residents’ exam have any value for the Society specialist title exam?	Yes	The 2025 initiative provides that, depending on performance, there will be a bonus for the TEOT theoretical test.	Not now. Under study	Yes	Yes, with a score.	Yes, depending on performance and attendance in the Progress Test, the resident can obtain exemption or bonus in the Theoretical Test of the Specialist Title in GO (TEGO).
Limitations not yet resolved	Discuss periodic revalidation	We believe that there is an adaptation in the Competency-Based Teaching model,	Maintenance of members of the Commissions, Adhesion to MR Programs, Title	Deepening in the evaluation of technical and non-technical skills and in simulation centers.	Introduction of practical tests	No
		with the implementation of the Matrix, which was updated by SBOT and needs to be endorsed by MEC. We are also developing models for categorizing the performance of residency programs, structuring a logbook, and developing APCs.	test litigations, financing of the PT			
Validity Time and Recognition of Title	No expiration date	It does not expire.	No renewal	Lifelong.	There is no validity and recognition time for the Specialist Title.	There is no revalidation or recertification
Revalidation/recertification of Title	There is no revalidation	No need to	No renewal	SBA Continuing Education Program. Certificate of Permanent Education in Anesthesiology (CEPE-A).	None.	There is no revalidation and recertification

*SBA - Sociedade Brasileira de Anestesiologia; SBOT - Sociedade Brasileira de Ortopedia e Traumatologia; CBO - Conselho Brasileiro de Oftalmologia; ABORL-CCF - Associação Brasileira de Otorrinolaringologia e Cirurgia Craniofacial; FBG - Federação Brasileira de Gastroenterologia; FEBRASGO - Federação Brasileira das Associações de Ginecologia e Obstetrícia.*
